# Independent Components of Neural Activity Carry Information on Individual Populations

**DOI:** 10.1371/journal.pone.0105071

**Published:** 2014-08-25

**Authors:** Helena Głąbska, Jan Potworowski, Szymon Łęski, Daniel K. Wójcik

**Affiliations:** Department of Neurophysiology, Nencki Institute of Experimental Biology, Warsaw, Poland; Georgia State University, United States of America

## Abstract

Local field potential (LFP), the low-frequency part of the potential recorded extracellularly in the brain, reflects neural activity at the population level. The interpretation of LFP is complicated because it can mix activity from remote cells, on the order of millimeters from the electrode. To understand better the relation between the recordings and the local activity of cells we used a large-scale network thalamocortical model to compute simultaneous LFP, transmembrane currents, and spiking activity. We used this model to study the information contained in independent components obtained from the reconstructed Current Source Density (CSD), which smooths transmembrane currents, decomposed further with Independent Component Analysis (ICA). We found that the three most robust components matched well the activity of two dominating cell populations: superior pyramidal cells in layer 2/3 (rhythmic spiking) and tufted pyramids from layer 5 (intrinsically bursting). The pyramidal population from layer 2/3 could not be well described as a product of spatial profile and temporal activation, but by a sum of two such products which we recovered in two of the ICA components in our analysis, which correspond to the two first principal components of PCA decomposition of layer 2/3 population activity. At low noise one more cell population could be discerned but it is unlikely that it could be recovered in experiment given typical noise ranges.

## Introduction

Local field potentials, the low-frequency part of the extracellular potential, are convenient signals to study activity of neural populations over temporal scales ranging from milliseconds to months [1], [2]. Easy to record, they are difficult to interpret, because the low frequencies of the potential can carry over long distances from a source [3]–[5]. As a result, every electrode picks a signal generated by a multitude of sources distributed over a substantial region.

In case of multielectrode recordings one may attempt reconstruction of current sources generating the measured potentials which helps to pinpoint the activity. Still, the obtained sources are superpositions of different overlapping populations. To extract activity of individual populations one can then use different techniques for signal decomposition, for instance independent component analysis (ICA [6], [7]) on which we shall concentrate in the present work, and indeed, success of several such approaches has been reported [8]–[10].

The challenge that remains is how can we be sure that the obtained components indeed carry functional meaning? Applying any algorithm to a dataset is bound to produce some results and the skill and the expert knowledge of the analyst are called for to justify their meaning. In particular, for the case of ICA, observe two issues: considering ICA a faithful model of the activity we assume the activity to be a sum of products of spatial profiles, 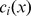, and temporal changes, 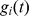: 
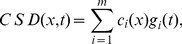
(1)which need not be true, at least for the small number of components we assume here. Secondly, we assume the sources to be statistically independent, yet we know that in the brain there is a strong coupling between different neural populations. It is thus far from obvious if the ICA is a feasible model for tackling the complexity of neural activity.

An ultimate test of any analytic approach is to analyze data for which the ground truth is known. The quality of the test with respect to its subsequent generalization depends on how realistic were the test data used. In the previous tests of the combinations of CSD analysis with component decompositions simple sources were typically used. For example, in [8] as the test data we used linear combinations of products of spatial sources and temporal profiles of the form (1). The coefficients 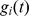 were functions of time of different classes: oscillatory functions (white noise low-pass filtered under 300 Hz), simulated evoked potentials and experimental evoked potentials. The spatial sources 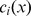 where constructed to resemble local CSD profiles observed in the studied experiment. While the obtained spatiotemporal activity often resembled experimental one, note that we imposed the structure of ICA on the test sources we used. Similar product sources were also used in [9]. More involved test data were employed in [11], where multiple copies of model data generated from activity of a single cell were used to achieve the level of a population signal.

Restricted nature of the sources used for tests so far has prompted some concerns regarding the validity of these approaches. For example, recently Gratiy et al. [12] wrote *“[…] PCA and ICA techniques decompose the signal into a sum of components with no reliance on the underlying biophysical processes and assume orthogonality or independence, respectively, of the processes to be isolated — assumptions that are likely to be invalid in the context of interacting neuronal populations.”* Thus, if we want to continue using these approaches, it is of utmost importance to find out to what extent the results of Independent Component Analysis — or any other competing method — can be interpreted functionally.

In this work we study the meaning of independent components obtained from CSD reconstructed with kCSD method [13] from a set of measured LFP. This is a method of LFP analysis we proposed in [8], the main modification being an improved method of source reconstruction (kCSD rather than iCSD). Our goal here is to use ground truth data generated with a complex network model of thalamocortical loop [14] to understand the functional meaning of components obtained from data accessible experimentally.

## Methods

### Extracellular potential and density of current sources

Extracellular potential in brain tissue is generated by transmembrane currents, mainly of the neural cells [1], [15]. A point current source 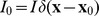 generates current density 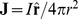, where 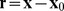, 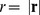, and 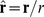. Using Ohm's law 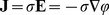 in a uniform and homogeneous medium we see that 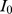 contributes a potential 
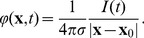
(2)


Contributions from multiple point sources 
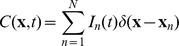
add up linearly
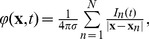
(3)and for a general distribution of current sources 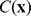 this formula generalizes to



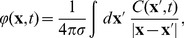
(4)A general relation between extracellular potential and the current sources is given by the Poisson equation 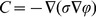
(5)which is also valid in more general media with inhomogeneous and anisotropic conductivity tensor 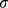.

If we know the distribution of the current sources, which is the case in simulations, we can use Eq. (4) to compute the potential in extracellular space. We call that *forward modeling*. In experiments we usually face the opposite: from the measured potentials we wish to extract the distribution of sources generating the potential using the Poisson equation (5). There are different numerical methods to achieve this, called generally Current Source Density methods. We call the problem of finding sources from potentials *inverse modeling* and we return to this problem in the next sections. For a more careful discussion of the relation of the extracellular potentials and current sources see for example [16], [17].

### A single column thalamocortical network model

**Figure 1:**
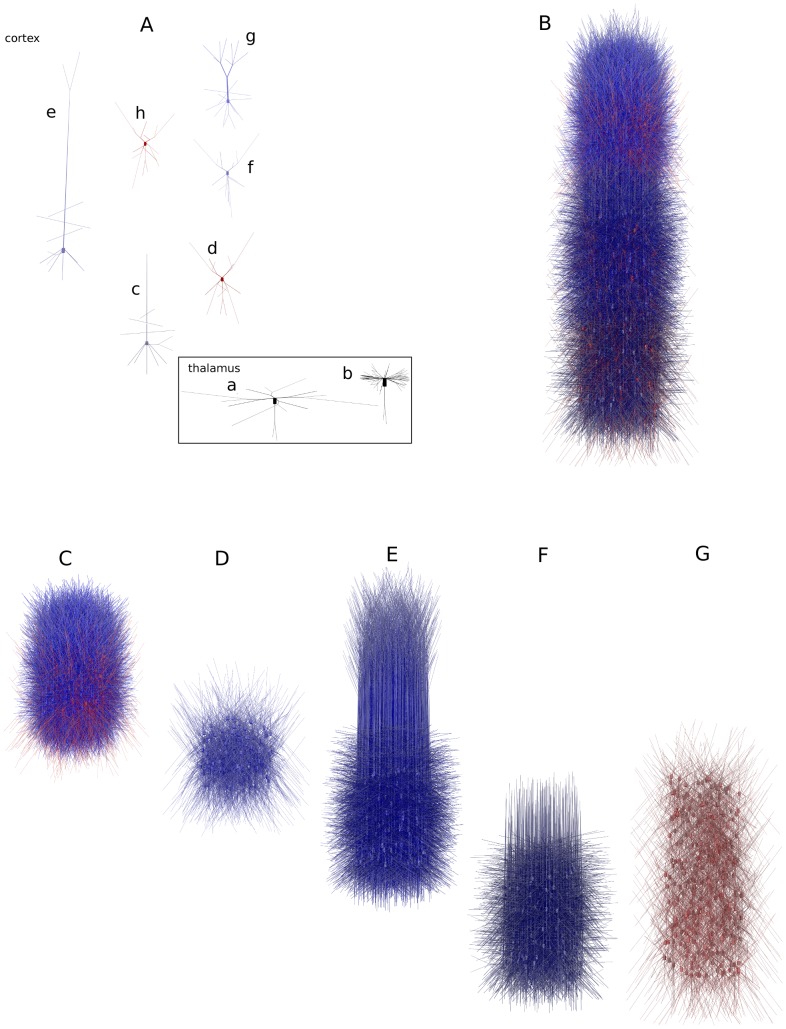
Structure of the model used. (**A**) **Morphologies of model cells a) nRT, b) TCR, c) layer 6 nontufted pyramidal, d) deep interneuron, e) layer 5 tufted pyramidal, f) layer 4 spiny stellate, g) layer 2/3 pyramidal, h) superficial interneuron (B) Cortical cells placed in cortical column, different populations are marked by different gradation of color (blue — excitatory cells, red — inhibitory cells).** (C–G) as (B) but separated into different layers: (C) layer 2/3, (D) layer 4, (E) layer 5, (F) layer 6, (G) layer 5/6.

To generate test data with realistic level of complexity we simulated a single-column model of thalamocortical loop based on Traub et al. (2005) [14]. Original version of the model was provided in IBM Fortran (ModelDB, accession number 45539). We based our study on versions in Neuron (ModelDB, accession number 82894) and neuroML (ModelDB, accession number 127353). This is the largest publicly available model of thalamocortical network. The default version contains 3560 multicompartment cells in 14 populations described in Table 1 and shown in Fig. 1. As in the original Neuron version every section contains a single segment.

**Table 1:** Cell types used in the model, numbers of sections in each cell and numbers of cells in each population.

Soma location	Population name	Number of sections	Number of cells
layer 2/3	pyramidal regular spiking (RS)	74	1000
layer 2/3	pyramidal fast rhythmic bursting (FRB)	74	50
layer 2/3	superficial interneurons — basket (bask), axoaxonic (ax) and low threshold spiking (LTS)	50	3 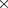 90
layer 4	spiny stellate (ss)	59	240
layer 5	pyramidal tufted intrinsic bursting (IB)	61	800
layer 5	pyramidal tufted regular spiking (RS)	61	200
layer 5/6	deep interneurons — basket (bask), axoaxonic (ax) and low threshold spiking (LTS)	59	3 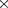 100
layer 6	pyramidal nontufted RS	59	500
thalamus	thalamocortical relay (TCR)	139	100
thalamus	nucleus reticularis (nRT)	59	100
total			3560

To model the extracellular potential we need a meaningful distribution of the cells and individual cell processes in space. To achieve that we took cell morphologies from the NeuroML version of the model [18] (Fig. 1 (A)) and distributed the neurons in a cortical column (Fig. 1 (B)). The somas of these cells were distributed randomly with a uniform distribution in particular layers within cylinders of radius 200* µm*. The depth of these cylinders for each layer is given in Table 2.

**Table 2:** Position of the cortical layers in the model.

Layer	Layer depth (*µ*m)	Populations
2/3	450–850	pyramidal RS, pyramidal FRB, basket, axoaxonic, LTS
4	850–1150	spiny stellate
5	1150–1650	pyramidal tufted IB, pyramidal tufted RS, deep basket, deep axoaxonic, deep LTS
6	1650–2150	pyramidal nontufted RS, deep basket, deep axoaxonic, deep LTS

### Calculation of model LFPs

Extracellular potential was computed under the assumption of homogeneous resistive medium. To calculate extracellular potential at a point 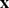 we used point source formula, Eq. (3), assuming point sources located in the center of every neural segment 
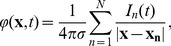
(6)here 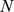 is the number of all neural compartments in the model, 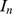 is the transmembrane current from the 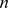-th current source positioned at 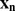, 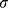 is the extracellular conductivity. We assumed 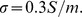 To get smoother data, more similar to what we observe experimentally, we calculated the random position of every cell 50 times. This led to denser network, less dependent on the seed. To obtain the LFP we low-pass filtered the computed potentials 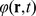 with a Butterworth second-order filter under 500 Hz using MATLAB.

We compared resulting LFP with those obtained using line source formula [19]: 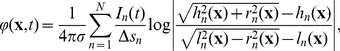
(7)where the additional variables 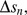 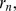 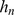 are the length of the 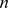-th line source, the radial distance from the source, the longitudinal distance from the end of the source and 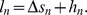 The difference between the results was negligible, but the computation took much longer so for all simulations reported here we used the point source formula.

### Simulations

**Figure 2:**
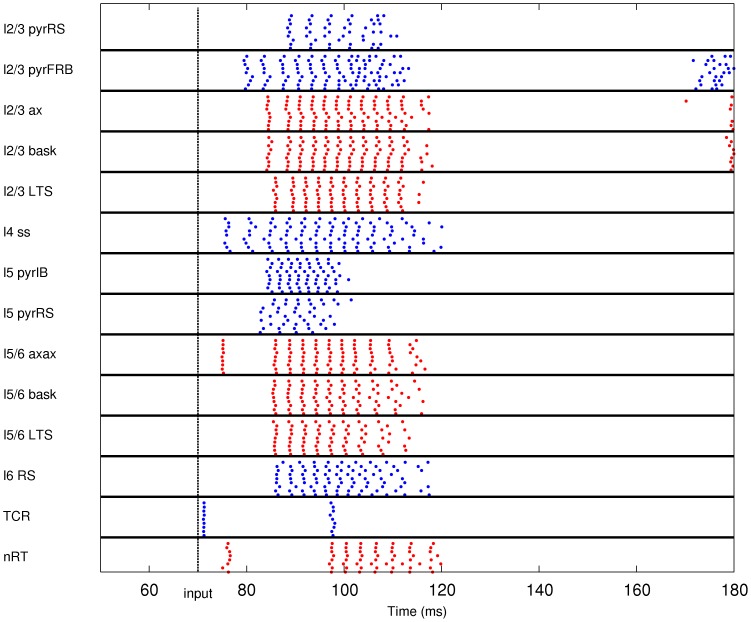
Raster plot of the network activity for the simulation of the response to whisker deflection. Blue dots indicate spikes of excitatory neurons, red dots indicate spikes of inhibitory cells. For clarity of the picture, only activity of ten cells from a given population is shown (activity of all cells within populations was similar). The first 50 ms of the simulation containing the turn-on artifact are not shown.

The simulations were ran in the NEURON simulator [20]. We considered two ways of stimulating the system. The first type of simulation was to model a response of a cortical barrel column to whisker deflection. Such input was simulated as injection of current into thalamocortical relay cells. A typical run consisted of 180 ms of simulation of network activity. During the first 50–60 ms the network shows transient turn-on behavior and then the spiking activity settles down. At 70 ms we injected a 3 ms long square current pulse of amplitude 2 nA into all of the thalamocortical relay cells. Such stimulus causes a few millisecond activation in thalamocortical relay cells, the activity then propagates to spiny stellate cells in layer 4, deep basket interneurons in layers 5–6 and nucleus reticularis in the thalamus. Then, the activation appears in fast rhythmic bursting cells in layer 2/3 and several milliseconds later in tufted pyramidal intrinsic bursting and regular spiking cells in layer 5, pyramidal regular spiking cells and interneurons in layer 2/3. Finally, the stimulus reaches the rest of the population in the cortex: nontufted pyramidal regular spiking neurons in layer 6 and the interneurons in layer 5/6. 50 ms after the onset of the stimulus the network calms down again (Fig. 2). To compute the LFP for the analysis in this case we used only the data from 50 to 140 ms.

**Figure 3:**
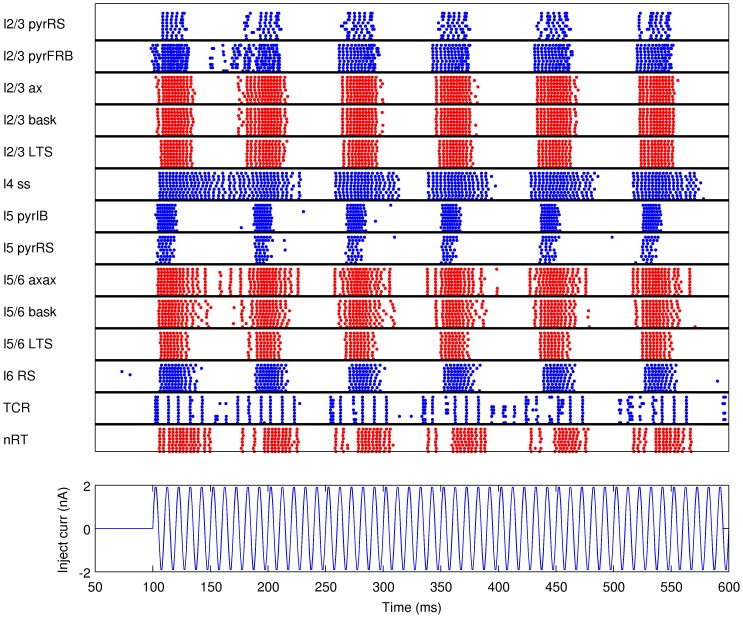
Raster plot of the network activity for the simulation of the response to injection of oscillatory current (100 Hz) to the thalamus. Blue dots indicate spikes of excitatory neurons, red dots indicate spikes of inhibitory cells. For clarity of the picture, only activity of ten cells from a given population is shown (activity of all cells within populations was similar).

In the second type of simulations the model was stimulated with injection of oscillatory current into the thalamic cells. We simulated 600 ms of network activity. At 100 ms after the onset of simulation a sinusoidal current with maximal amplitude of 2 nA was injected into all of the thalamocortical relay cells, which caused oscillatory response in the cortex (Fig. 3). The frequency of the input current was 12.5 Hz, 25 Hz, 50 Hz, 100 Hz, or 200 Hz in different runs.

In all the simulations the gap junctions and ectopic spikes were disabled in the model [14]. There were several reasons for turning off gap junctions. First, the Neuron implementation of the Traub's model was never tested with gap junctions. Secondly, inclusions of gap junctions in the model makes the calculation time much larger (which could be the reason for lack of thorough tests). Thirdly, we were not able to run the model with gap junctions with variable time step (needed for precise transmembrane currents estimation) and in parallel mode (needed to get the results in a reasonable time) on version 7.1 or older, and in Neuron 7.2, at least on BlueGene Q. Finally, the place where the gap junctions appear to influence model dynamics the most is the coupling between pyramidal cells, which by itself is a controversial hypothesis.

To compute LFP, the sum of all transmembrane currents from every segment was saved every 0.1 ms.

### Datasets

In total, we analyzed six datasets, one obtained through modeling of the whisker deflection, and five obtained through simulating injection of oscillatory input current with different frequencies (see above). Each dataset consisted of twelve matrices 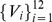 containing the values of LFP generated by individual populations of cortical neurons at the assumed positions of the electrodes, and so their sum 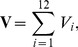
(8)contained the total LFP generated by the whole column, which would be the only signal accessible in the experimental setting. The size of all the matrices was 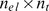, where the number of electrodes 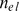 was 26 positioned along a vertical line every 50* µ*m, and the number of samples 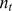 was 900 for the whisker deflection data, or 5000 for the oscillatory data.

The density of the current sources for each cell population, 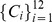, was represented by the values estimated from the potentials using kernel CSD method (see the next section). This procedure was chosen to reproduce the analysis which would be applied in genuine experimental context. A relation of this representation of CSD to the actual transmembrane currents in the population is discussed later on. The complete simulated activity was represented by reconstruction from the summary LFP: 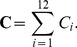
(9)


The CSD was reconstructed in 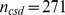 points distributed regularly in space spanning position from 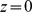 to 2700* µ*m using 1D kCSD.

### Current Source Density reconstruction

Observing the relation between the extracellular potential and the current sources, Eq. (5), Pitts [21] proposed to estimate CSD using the finite-difference approximation. If we consider a laminar multielectrode, where the contacts are uniformly spaced: 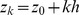, inserted in the cortex perpendicularly to the laminae, and assume uniform potential along the layers, the CSD can be approximated as follows [22]: 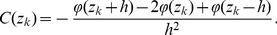
(10)


We call this approach Traditional CSD Method. The deficiencies and problems of this straightforward approach have been discussed recently and a number of alternative estimation methods were proposed [13], [23]–[26].

Here we use Kernel Current Source Density (kCSD) method [13] which for one-dimensional estimation works as follows. Since we do not know the values of the sources (potentials) in the directions we do not measure we assume the sources to be constant on discs of radius 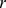 orthogonal to the line of measurements and consider a class of CSD distributions of the form 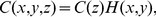
(11)where
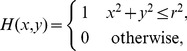
(12)for 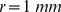. We also assume here that the conductivity 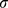 is constant, so the medium is isotropic and homogeneous. We construct the one-dimensional profile 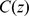 from a basis of functions 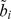 densely covering the line. These can be step or Gaussian functions
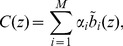
(13)where 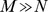, 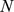 being the number of electrodes. Substituting Eq. (11) and (12) into (4) we find the potential profile generated by (13) to be



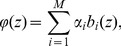
(14)where 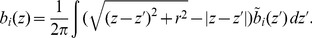
(15)


Define kernel 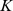 and cross-kernel 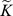 functions as 
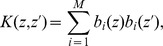





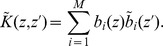



In the noise free case one can show [13] that among the multiple functions of the form (14), which satisfy 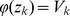, the one that minimizes the norm 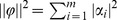 is given by 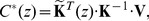
(16)where 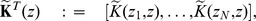





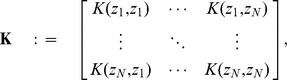





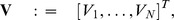
where 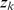 are the positions of the contacts, and 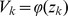 are the measured values of the potentials. Finally, to avoid over-fitting to noisy data one can use ridge regression obtaining the form of kCSD we use here:



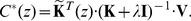
(17)For details see [13].

### Independent Component Analysis

Consider multiple simultaneous recordings of linear mixtures of signals generated by several sources. A common example is recordings of speech of two people with two microphones. If the sources are statistically independent one can use independent component analysis (ICA) to recover the source signals [6], [7]. In our context, extracellular potentials measured (simulated) are contributed by different cells. While we cannot assume independence of cell activity within a population, we show here that even strongly coupled populations generate signals which are sufficiently independent to warrant a meaningful decomposition. In this work we apply the ICA to CSD reconstructed from the simulated potentials (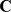) and study to what degree the decomposition in Eq. (9) can be recovered. We have tested temporal, spatial and spatiotemporal ICA [27].

#### Principal Component Analysis

The first step of ICA analysis is dimensionality reduction obtained through Principal Component Analysis (PCA). PCA projects the data linearly onto a subspace preserving as much information as possible and getting rid of assumable noise. Consider the SVD factorization [28], [29] of the 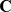 matrix: 
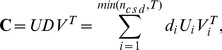
(18)where 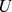 is of size 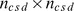 and its columns are called eigenimages, 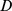 is a diagonal matrix of size 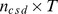, whose diagonal values 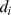 are called singular values and V is of size 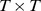 and it's rows are called eigensequences. The idea behind PCA is to approximate 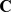 with a truncation of the (18) keeping only the terms corresponding to the 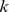 (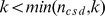) largest singular values 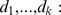




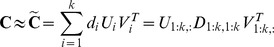
(19)To simplify this, we define: 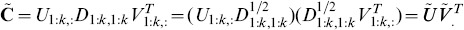
(20)


Each element of the sum in (19) is called a principal component. Choosing the number of principal components 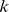 correctly is important as it also determines the number of independent components the ICA algorithm will produce.

#### Spatial ICA

Spatial ICA (sICA) is based on the assumption that each image in 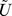 from Equation (20) is a linear combination of 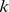 spatially independent images: 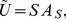
(21)where S is a (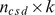) matrix whose rows are independent image vectors. If we knew 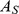 we could obtain the independent image vectors by computing 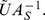 We choose it by first assuming a probability distribution for the independent image vectors. Then we decide on the 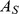 that maximizes the entropy 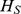 (see [6], Chapter 7.3) of 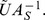 computed under this probability distribution. From (20) and (21) we see that 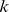 unconstrained dual time courses can be obtained by computing 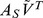.

Spatial ICA can perform well in situations where there exist spatially organized modules that perform distinct functions and are generators of independent signals. An example can be the analysis of fMRI data [30] or of averaged stimulus-evoked potentials, where the signals are related to specific brain computation performed by a network with fixed anatomical connections [8].

#### Temporal ICA

Temporal ICA (tICA) is conceptually dual to sICA. We assume here that each sequence in 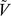 from Equation (20) is a linear combination of 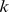 independent temporal sequences: 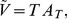
(22)where T is of size (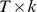). Again, we assume some probability distribution for the temporal sequences (columns of T) and choose 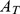 that maximizes the entropy 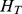 of 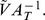 The 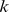 unconstrained dual images can be obtained by computing 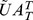.

In terms of neural data analysis temporal ICA assumes independent processes taking place in the brain at the same moment. It seems to work well with EEG data [31]–[34]) where one can consider the resulting signals to be produced by independent generators performing different actions simultaneously.

#### Spatiotemporal ICA

Spatiotemporal ICA (stICA) is based on an assumption about independence both throughout space and time. Namely it is assumed that 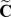 can be decomposed 
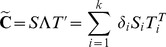
(23)where 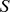 is a 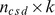 matrix of 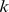 mutually independent images, 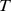 is an 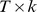 matrix of mutually independent time sequences and 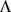 is a diagonal scaling matrix. It is also assumed that there exist two mixing matrices 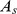 and 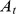 such that 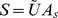 and 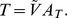 Since
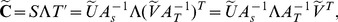
(24)it follows that 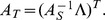


We assume probability distributions for the independent image vectors and temporal sequences separately. As in the previous cases for given mixing matrices 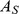 and 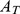 the spatial and temporal entropies 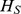 and 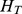 can be calculated. The principle behind stICA is to maximize a linear combination of the entropies: 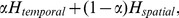
(25)where 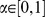 quantifies how much weight we attribute to the temporal and spatial independence. Thanks to (24) we see that the maximization can be run over 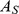 and the diagonal matrix 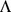. stICA seems to to perform well in fMRI data analysis [27] and in analysis of LFP data from multielectrodes [8].

To analyze the data from the Traub model we applied sICA and stICA. We used the MATLAB code (STICA software available at http://jim-stone.staff.shef.ac.uk) by J.V. Stone and J. Porrill. We imposed conditions of high curtosis of the spatial independent components, which can be done assuming the probability density function (pdf) of the form 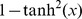. The pdf for the temporal signals was assumed of the form 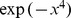 (a low-curtosis distribution). Those were the same choices as in [8].

### Density of current sources computed directly from the model vs. through kCSD from the LFP

**Figure 4:**
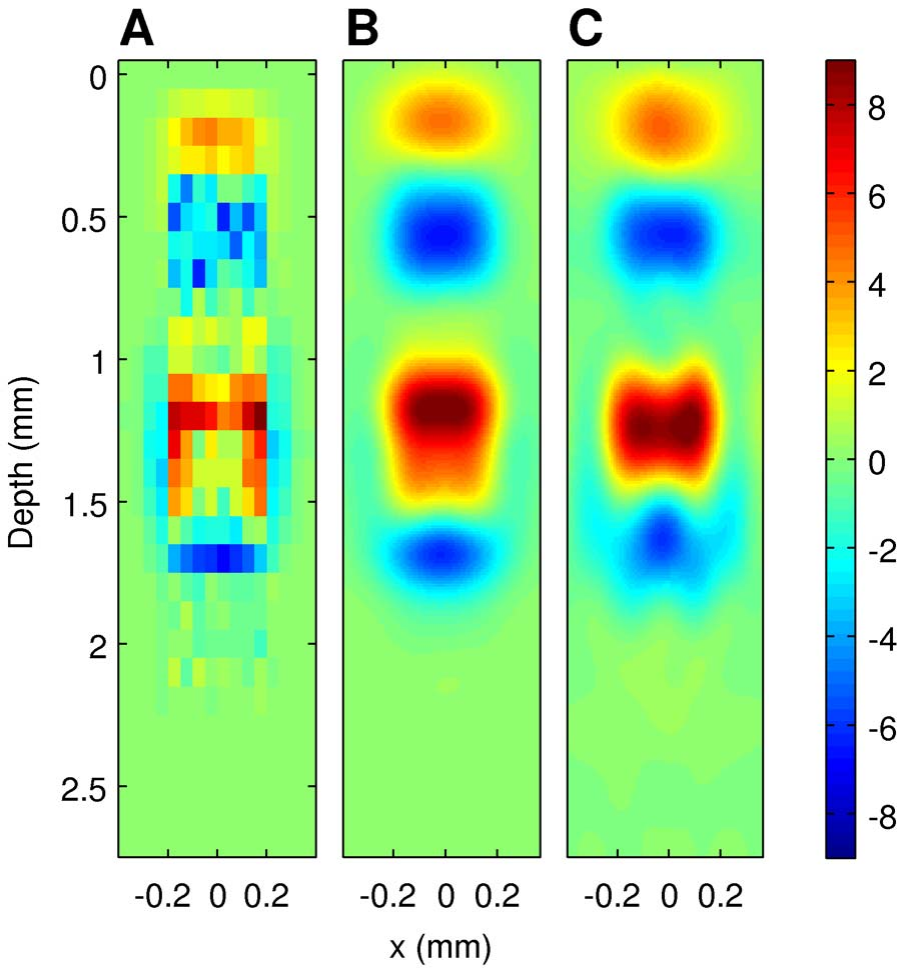
Different representations of CSD. A) current sources obtained in a simulation summed within boxes of [50* μm*×100* μm*×50* μm*] in the plane cutting through the axes of the column on which virtual electrode contacts were positioned. B) coarse-grained CSD (smoothed with a Gaussian kernel of 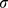  = 80* µm*) through the same plane, and C) a reconstruction with kernel CSD method from LFP computed at a grid of 8×14 electrodes from the full simulation data. Vertical distance is given in *µ*m from cortical surface, horizontal from the center of the simulated column.

In the present work as the reference representation of ground truth current sources (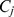 in Eq. (28)) we took the CSD reconstructed from simulated contributions to the LFP coming from given cell population. One may wonder to what extent the selected procedure of computing current sources from potentials rather than directly influences our interpretation of results. We have previously compared the shape of sources reconstructed from potentials against the true sources which generated them, in the case of assumed smooth sources [13], [24], [26]. In the case of the Traub's model we have a sparse distribution of the sources in space. What is physiologically meaningful is the coarse-grained density of the current sources in the space. To compare the different representations of CSD, Fig. 4 shows A) current sources obtained in a simulation summed within boxes of [50* µm*×100* µm*×50* µm*] in the plane cutting through the axes of the column on which virtual electrode contacts were positioned; B) coarse-grained CSD (smoothed with a Gaussian kernel of 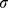  = 80* µm*) through the same plane, and C) a reconstruction with kernel CSD method from LFP computed at a grid of 8×14 electrodes from the full simulation data. Vertical distance is given in *µ*m from cortical surface, horizontal from the center of the simulated column.

The coarse-grained CSD in Fig. 4 B) was calculated as a running average of computed currents with Gaussian kernel 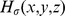: 
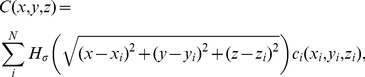
(26)where 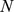 is the number of all the current sources, 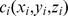 is the current source placed at 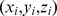, and
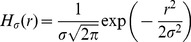
(27)for 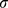  = 80* µ*m. While some differences between B) and C) are apparent, overall we can see that CSD reconstructed with kCSD reflects the coarse-grained sources well, which is how the reconstructed CSD should be understood.

## Results

### Recovering activity of individual cell population in noise-free case

The goal of this work was to study the relation of the independent components of CSD reconstructed from (simulated) measured potentials to the activity of specific cell populations. We first simulated the network, and computed extracellular potentials as described in the Methods section, which gave us ground truth data — simulated CSD of individual cell populations — as well as a set of generated recordings akin to what could be obtained experimentally.

**Figure 5:**
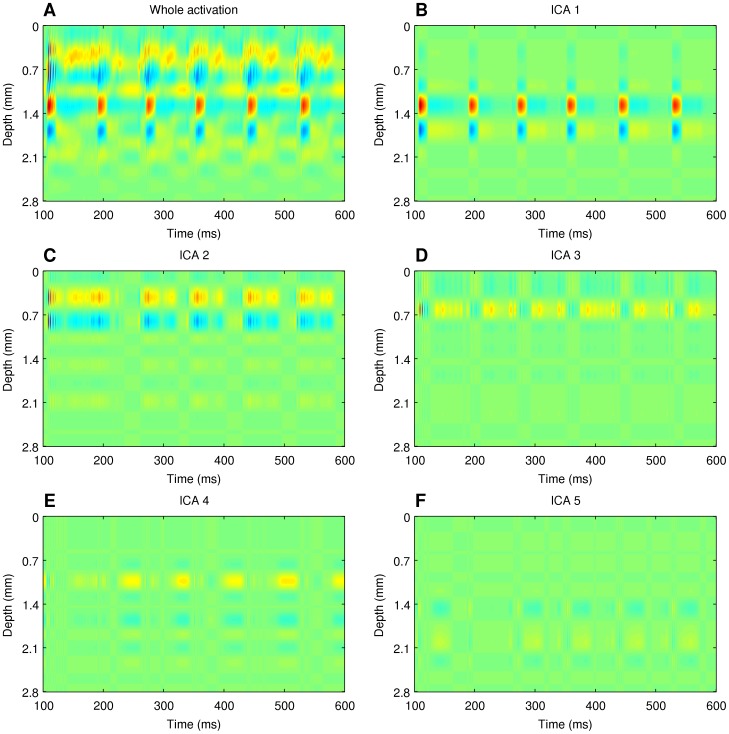
Independent components of reconstructed CSD. (B)–(E), ICs obtained in the spatial decomposition of the CSD reconstructed with kCSD method from the simulated potentials, (A). Red — source (positive CSD), blue — sink (negative CSD). This color code is kept in the whole article

We then analyzed the simulated potentials as we would the experimental recordings, that is, we first reconstructed current sources from them using the kCSD method (see Methods for details). Fig. 5A shows the total reconstructed CSD through the center of the column as a function of time for the example data obtained for 100 Hz oscillatory stimulation of the thalamus (see Methods for the description of datasets used). Then, we reduced the dimensionality of the obtained reconstruction 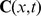 using PCA, and decomposed it using ICA. We considered both spatial and spatiotemporal ICA as described in the Methods. Since the results for spatiotemporal ICA decomposition were not better than for purely spatial ICA decomposition, we describe here only the results for the latter case. Fig. 5B–F shows a spatiotemporal representation of five independent components obtained in spatial ICA decomposition of this dataset. Each plot here shows a product of the spatial profile 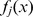 and the time course 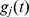 defining a given component 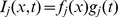, where 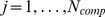, with 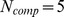 here.

**Figure 6:**
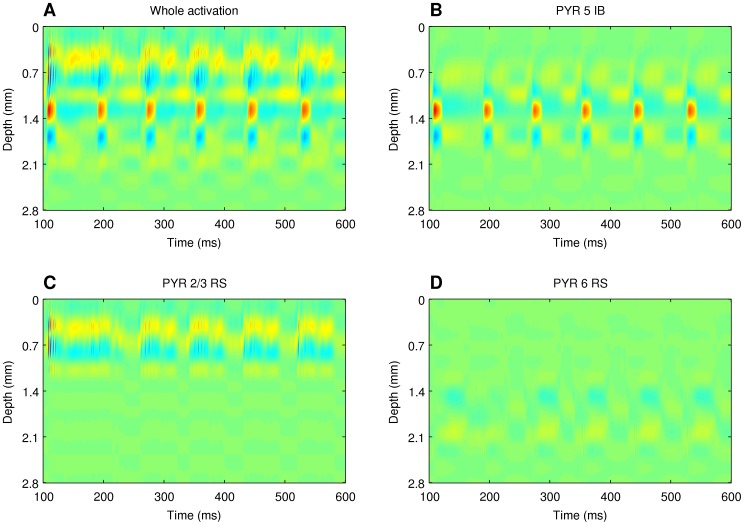
CSD of individual populations. Current source density of the whole network A), and contributions from individual cell populations B), C) D).

To understand the meaning of the resulting components we compared them with the CSD reconstructions representing individual population activity, Eq. (9): 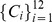. Fig. 6B–D shows the reconstructed CSD of the three population giving the largest contributions to the total activity, Fig. 6A. Those are pyramidal ‘intrinsic bursting’ cells from layer 5 (B), pyramidal ‘regular spiking’ cells from layer 2/3 (C), and pyramidal ‘regular spiking’ cells from layer 6 (D).

The product nature of the independent components is clearly visible in Fig. 5 and noticeably different from the activity of individual populations, Fig. 6. A comparison of these figures immediately reveals that one cannot expect ICA analysis of such complex data to recover population activity as single components. To find out how the independent components relate to individual populations we considered all possible distributions of the components into groups. To find out how well a given subset of ICs describes the activity of a selected population 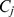 for 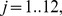 we compared all linear combinations 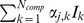, where 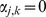 or 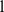, and 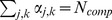 (each component assigned to only one population), with the reconstructed activity of every population 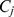, using correlation as a measure of similarity 
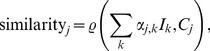
(28)where 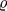 denotes the Pearson's correlation coefficient 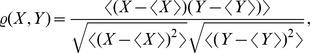
and where 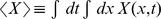.

For every studied data set we found that it is possible to recover reliably activity of two cell populations, pyramidal cells in layer 2/3 and layer 5, with correlations of 0.92 and 0.88 respectively for the 100 Hz stimulation (on average, for all datasets: 0.91 and 0.9). In all cases but one also the activity of pyramidal cells from layer 6 could be obtained (0.70 in the case of 100 Hz stimulus, 0.74 on average; see Table 3). To get this result we need 2 independent components (ICs) for pyramidal cells in layers 2/3, either 3 (50 Hz dataset) or 2 (all other datasets) ICs for pyramidal cells in layer 5, and 1 IC for pyramidal cells in layer 6 (in cases where this population could be identified at all).

**Table 3:** Correlation between independent components obtained through our procedure with activity of specific cell populations for different example simulations.

Dataset	PYR 2/3 RS	PYR 5 IB	PYR 6 RS
whisker deflection	0.95	0.91	-
12,5 Hz	0.95	0.9	0.68
25 Hz	0.88	0.93	0.79
50 Hz	0.83	0.91	0.76
100 Hz	0.92	0.88	0.70
200 Hz	0.94	0.9	0.76

**Figure 7:**
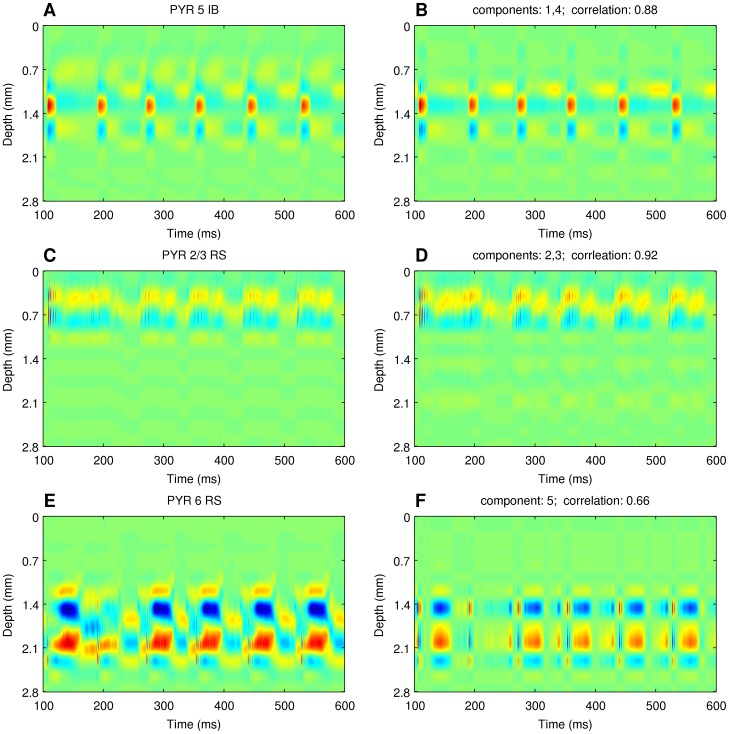
Recovering activity of population from independent components. Comparison of the spatiotemporal profiles of the activity of the three populations dominating the CSD with the profiles of summed independent components for the case of Figure 6. Note that the color scale on the figures has been set the same for every row and is selected to emphasize each activity.

Figure 7 compares the spatiotemporal profiles of the activity of the three populations dominating the CSD with the profiles of summed independent components for the case of data shown in Figure 6. These results show that applying the proposed method of analysis (reconstruction of current sources from LFP through kCSD followed by spatial ICA and grouping the components) to simulated cortical LFPs allows one to obtain a good approximation of the activity of the individual cell populations of pyramidal cells (layer 2/3 and layer 5) with less reliable recovery of the activity of pyramidal cells from layer 6. The contributions from the other populations to the CSD and LFP are too weak to be recovered from recordings, even though their role in setting the network activity, synchrony, etc. may be important.

#### The influence of spatial relation on the quality of reconstruction

In the original model the spatial distributions of the populations contributing the most to the simulated recordings, layer 5 and layer 2/3 cells, were quite separated in space. One may wonder what will happen if we shift the two populations with respect to each other. While the network dynamics does not change with such an exercise, the observed potential and the spatial profiles of the CSD would differ.

**Figure 8:**
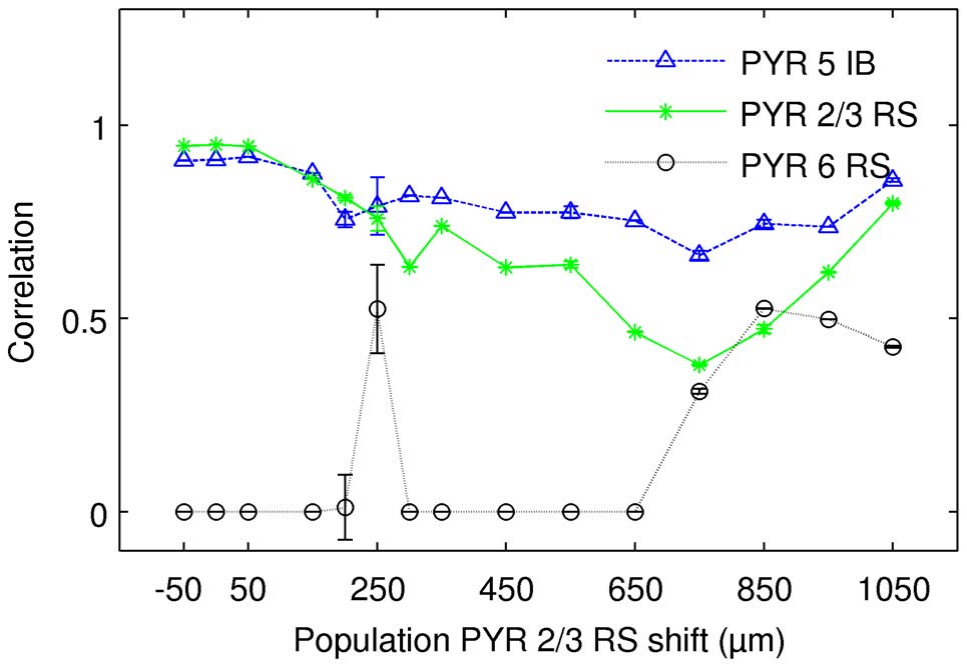
Quality of reconstruction of the three dominating populations for data generated by shifting population of layer 2/3 cells downwards. Observe that the quality of reconstruction for the two main populations remains rather stable while the reconstruction of the third population becomes discernible only for some locations. Data from the simulation of whisker deflection. Results of 50 repetitions of ICA algorithm, error bars denote standard deviation.

Fig. 8 shows quality of reconstruction of the three dominating populations for data generated by shifting population of layer 2/3 cells downwards in the simulation of whisker deflection. Observe that the quality of reconstruction for the two main populations (layer 5 and layer 2/3) remains rather stable although it drops slightly, more for layer 2/3 cells, as we move them down. At some point the third population (layer 6 cells) becomes discernible.

It is difficult to understand which features of the data can lead to possibility of recovering of the third population, in particular the third population appearing for the shift of 250* µ*m. To make sure this result is not an artifact of, say, stochastic aspects of the ICA algorithm, we repeated this analysis 50 times for every point in this plot. As we can see, the quality of the reconstruction of the third population at this point has some variability, as marked by the std bar, but we could always reconstruct here the three populations. For all the other points the results are very stable.

#### The influence of the number of electrodes on reconstruction

**Figure 9:**
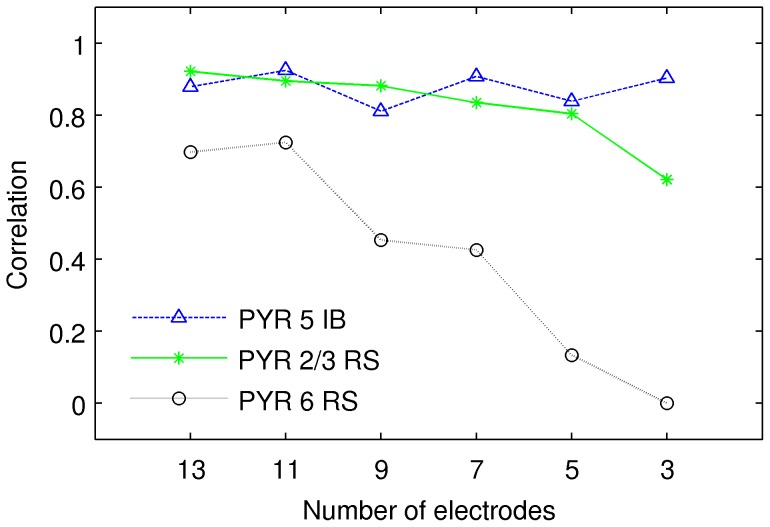
Quality of reconstruction of individual cell populations from decreasing number of electrodes. Quality was judged against equivalent representation of individual cell populations.

Given that we can reconstruct at most three populations anyway, one may wonder how many recordings need to be taken to perform the decomposition. We have repeated our experiments for the 100 Hz stimulus selecting for analysis subsets of 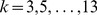 simulated recordings from equally distant electrodes selected so as to span the largest possible extent of the total considered depth. We repeated the procedures of CSD reconstruction followed by the ICA decomposition. We compared thus obtained components with CSD of individual populations represented in the same way, that is by CSD reconstructed from LFP taken at the same recording points. We found that while the third population becomes less and less discernible with decreasing number of electrodes, the two main populations remain remarkably stable (Fig. 9).

### The influence of noise on reconstruction

**Figure 10:**
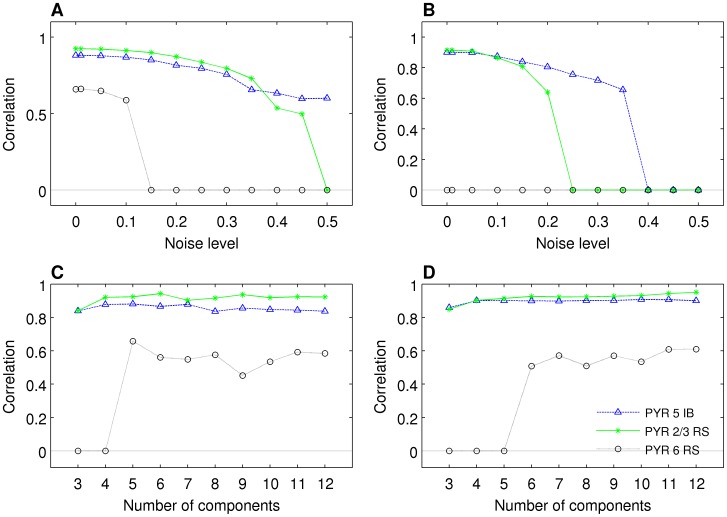
Recovering activity of populations for different noise levels. Simulations with A) 13 electrodes, B) 26 electrodes; and for varying numbers of components assumed in ICA: simulations with C) 13 electrodes, D) 26 electrodes. X-axis: A), B), noise level, see text for details, C), D), number of components; y-axis: correlation of the recovered population activity with the original population activity for optimal choice of components. The value of 0 denotes cases where the activity of the original population correlated better with a constant, zero-valued signal, than with any combination of components.

All the results so far were obtained in the ideal case with no measurement noise which does not happen in reality. So the natural question is how these results need to be modified in the realistic case with significant noise. It turns out, that the noise in fact does not degrade the results significantly. Thus, while in the experiment we may not be able to observe more than 2–3 dominating cell populations, their recovery is rather robust. Fig. 10A, B show the values of the correlation coefficients between the three dominating populations and the best selection of independent components as a function of noise level in the data. To obtain this figure we simulated white noise with amplitude scale set as follows: the values of all the potentials for all times were pooled and standard deviation of their distribution was computed. This was used to set the amplitude (100%) of the additive noise added independently on every electrode. As we can see, the dominating two populations can be recovered even for significant noise (up to 50%), while the third strongest population cannot be recovered for noise levels exceeding 15%.

We have also studied the influence of the number of components 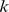 assumed in the ICA algorithm on identification of population, Figure 10C, D. Note that we need at least five or six components to be able to recover the third strongest population, PYR 6 RS.

Note also that correlation levels shown in 10 were obtained with prior knowledge of the ground-truth signals, which allowed us to combine the right components together; for that reason they should be interpreted as upper-bounds of reconstruction quality. The question of how to deal with this issue in case of experimental data is discussed in the final section.

### Relation of the independent components of the total activity to the principal components of individual populations activity

**Figure 11:**
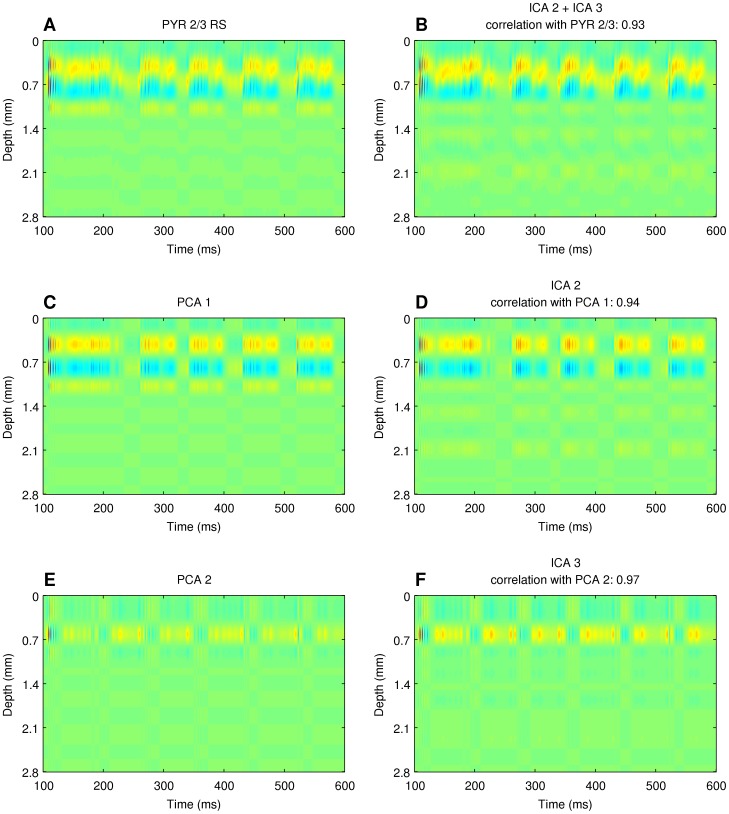
The meaning of independent components of total activity. Comparison of independent components of the total activity (B), (D), (F) to the principal components of individual populations activity, (A), (C), (E).

One puzzle still remaining is what is the relation of the independent components obtained above, 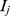, to the individual cell populations activities, 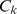. To understand this relation better we performed PCA decomposing 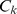. We found that the independent components of the total CSD signal reflect well the principal components of individual cell populations. Figure 11 shows an example comparison of the strongly non-product activity of the population of pyramidal cells from layer 2/3 with the sum of two independent components, and compares the respective ICs with the principal components of this population activity.

## Discussion

By combining modeling of extracellular potentials in a large-scale model of thalamocortical loop with data analysis employing kCSD and ICA we showed that:

- Spatiotemporal activity of model populations may not have simple product structure, hence it may not be possible to represent activity of a population with just one product component of the kind typically assumed in ICA or other decomposition methods (Fig. 7).
- Independent components recovered using ICA correspond to principal components of the true activity of individual populations, rather than to the full activity (Fig. 11).
- LFP activity of a cortical column is dominated by the contribution from pyramidal cells (Fig. 6).
- The combination of kCSD and ICA allows to recover activity of two strong populations of pyramidal cells (PYR 5 IB, PYR 2/3 RS) and, in most cases, one weaker (PYR 6 RS) population (Fig. 8).
- These results are robust against relative spatial positions of these populations, against noise and are preserved for small numbers of electrodes and components (Fig. 9, 10).

Our results lead us to conjecture that the proposed method of analysis: reconstruction of current sources from recorded LFP followed by spatial ICA and grouping the components, allows one to obtain a good approximation of the activity of individual cell populations contributing to the extracellular potential. In the cortex, these will typically be large populations of pyramidal cells, however, depending on the size of the population and their synchrony, as well as the composition of the network in the studied region of the brain (e.g. thalamus), cells with more closed fields, such as stellate cells, for example, may also have sizable contributions [4],[5], which could in turn be recovered from data [8].

The obtained results carry both good and bad news. The bad news are that even with substantial number of electrodes one cannot recover more than three populations out of twelve. On the other hand, the good news is that top two populations are very robust and even with substantial amount of uncorrelated noise on top of the measurements, for just a few electrodes, one can reliably recover their activity with kCSD-ICA. The robustness of these signals, of course, lies at the heart of the utility of extracellular electrophysiology of slow potentials. What we show here goes beyond traditional approaches as we can identify activity of individual cell populations, and as such give functional meaning to the results of previous analyses [8].

One aspect of the proposed scheme for identification of activities of individual cell populations is finding the ICs which need to be combined. We have tried a number of data-based schemes, including correlating different spatial and temporal components, and we have not been able to find one which we could recommend as completely general working in every situation. This can be easily understood: Since, as we show, ICs describing a given population are principal components of this population activity, which are by definition orthogonal, their correlation should be zero. Moreover, if we have coupled populations, they would typically drive one another with similar frequency content in both, thus degrading the usefulness of frequency based schemes. Even after smoothing spatial profiles and demanding substantial overlap between two spatial IC profiles we see that the components corresponding to different but spatially overlapping populations would typically overlap too.

This conundrum is difficult to solve using insights coming purely from data. In our view, there are two solutions to recommend in analysis of experimental data, where the ground truth is unavailable: 1) judging by the prior anatomical and physiological knowledge of the studied system one must postulate the possible position and number of relevant cell populations and then try to match the obtained components until plausibility is obtained; 2) use the insights from analysis of model data, coming from models of the studied system, to judge what are the possible shapes of components to expect. We believe that even with crude models of the system this scheme might be efficient. For example, our expectation for the analysis of experimental cortical data from different areas would be to run such analysis for at least 10 contacts, using at least 5 ICA components in spatial ICA, and then match them according to the position of the spatial part.

We do not exclude the possibility that there might be a viable data-based solution for identification of the components to match, however, we have not been able to identify one.

In a way, our previous study [8], from the perspective of ICA validity was performed on simpler data, in the sense that in the thalamus the coupling between individual cells is indirect (driven by the sensory inputs and cortical feedback). We believe that the strongly coupled network modeled here provided much more realistic model of the experimental cortical LFP and as such provides a much stronger validation of the proposed analytic approach than the previous studies.

Since the two elementary operations used here (CSD, ICA) formally do not commute, one may wonder if their order matters in practice. In their studies, Makarov, Herreras, et al. [9], [11] report positive results for the approach when they first decompose the signals and then reconstruct the sources. While the results may also depend on the data being analyzed, our experience when we tried both approaches [8] is that recovering sources before doing decomposition is generally a more beneficial strategy. Conceptually, CSD is sharpening the picture of neural activity restricting the extent of individual sources. As such it seems to us a good preprocessing step for further decomposition.

In analysis of experimental data one has to take into account the presence of inherent noise. The kernel CSD method we use here [13] is to our knowledge the only technique for CSD estimation from incomplete array of electrodes that avoids overfitting to noise which is why we also use and recommend it. Another aspect, possibly degrading the quality of the results obtained experimentally, is that in real brain any column is surrounded by neighbors contributing to the measured potential. This is where the CSD analysis is helpful, as it recovers the local sources from the nonlocal potentials.

In this work we studied the performance of ICA in extracting activity of individual cell populations but there are other methods of decomposition. Di and Barth [35] used principal component analysis to indicate the dominating contributions of the supra- and infragranular pyramidal cells to the recorded LFP in the barrel cortex. While the PCA gives useful results, we believe ICA is a more powerful method and may provide richer output.

Another technique introduced recently is Laminar Population Analysis [10]. The approach here is different than in PCA and ICA, namely one assumes a specific set of populations and considers their connectivity matrix. Then, using the data on the pooled inputs (CSD) and outputs (multi-unit activity, MUA) of the populations one fits the coefficients in the modeled equations to obtain information on the relevant populations. While this method may potentially provide deeper results it also requires more data (MUA as well as LFP). Also, it has not been validated with similarly complex data for which ground truth is known. A comparison of the method presented here with LPA studied for the same model data is planned for the future.

In this paper the LFP is computed from the currents using assumptions of uniform and homogeneous medium. While the brain tissue is inhomogeneous and anisotropic, recent results for microscale inhomogeneity [36] do not indicate the need for modifications of Eq. (4) at the relevant spatial and temporal scales. Also, the cortical anisotropy studied for example in the rat [37] seems meaningless to include in the modeling, since the variability in the measured values of conductivity tensor across specimen is comparable with observed anisotropy. While the specific contributions from different biophysical mechanisms to different aspects of LFP are highly debated today [1], [38], [39], we believe that the approach we use is still adequate, indeed, the most appropriate for estimation of extracellular potential in simulations [2].
